# Anterior and Posterior Cruciate Ligaments Mechanoreceptors: A Review of Basic Science

**DOI:** 10.3390/diagnostics12020331

**Published:** 2022-01-27

**Authors:** Konstantinos Banios, Vasileios Raoulis, Apostolos Fyllos, Dimitrios Chytas, Vasileios Mitrousias, Aristeidis Zibis

**Affiliations:** 1 Department of Orthopedic Surgery, General Hospital of Karditsa, Peripheral Road Karditsa-Kastania, 43100 Karditsa, Greece; konmpanios@hotmail.com; 2 Laboratory of Anatomy, Department of Medicine, School of Health Sciences, University of Thessaly, 3 University Street, Biopolis, 41110 Larissa, Greece; v_raoulis@yahoo.gr (V.R.); apofyl@hotmail.com (A.F.); vasileiosmitrousias@gmail.com (V.M.); 3 Department of Physiotherapy, University of Peloponnese, 20 Plateon Street, 23100 Sparta, Greece; dimitrioschytas@gmail.com; 4 Department of Orthopedic Surgery, University of Thessaly, 3 University Street, Biopolis, 41110 Larissa, Greece

**Keywords:** mechanoreceptors, proprioception, anterior cruciate ligament, posterior cruciate ligament, knee joint

## Abstract

Proprioception is a specialized sensory modality encompassing the movement of the joint and its position in space, and it involves the conversion of mechanical deformation of tissues into neural signals. Mechanoreceptors are specialized nerve structures able to transmit mechanical deformation through electrical signals to dorsal root ganglion sensory neurons and are abundant in the muscles, tendons and ligaments of the knee joint. They are believed to play an important role in knee proprioception and dynamic knee stability. Proprioception should always be taken into consideration for successful reconstruction of the cruciate-deficient knee and for pain and function management in the arthritic knee. Advances in histological methods of detection are numerous and continue to highlight the presence and role of mechanoreceptors after ligament reconstruction, depending on choice of graft. In this review, we present the current knowledge of anterior and posterior cruciate ligaments and grafts mechanoreceptors, and their role in proprioception of knee joint, focusing on each type of mechanoreceptors.

## 1. Introduction

The knee joint is comprised of a complex configuration of osseous and soft tissue structures that work in conjunction to allow three planes of motion. The static stabilizers of the knee are its ligaments. The main restraints of tibial translation relative to femur are the cruciate ligaments. In particular, the anterior cruciate ligament (ACL) prevents the anterior translation and the posterior cruciate ligament (PCL) prevents the posterior one, acting as a counterpart to the ACL.

Both the anterior and posterior cruciate ligaments play an extremely significant role in knee joint stability. Apart from being the main restraint against anterior or posterior translation of the tibia relative to the femur, they also contribute to dynamic stability of the knee joint via proprioception and activation of knee muscles [1]. The presence and function of mechanoreceptors (MRCs) that are responsible for proprioceptive function in the ACL and PCL have gained interest recently. Several studies have demonstrated that MRCs are present in both ligaments [2,3,4,5,6].

The aim of this review is to present the current knowledge of anterior and posterior cruciate ligaments mechanoreceptors and their role in the knee.

## 2. Proprioception—Basic Science

Proprioception is a specialized sensory modality encompassing the movement of the joint and its position in space. There are three main functions of proprioception:

1. Static awareness of joint position;
2. Awareness of joint movement and acceleration;
3. Reflex responding and regulating muscle activity.

Moreover, proprioception plays an important role in preventing injuries and maintaining function of the knee joint [2]. The sense of proprioception involves MRCs, which are specialized nerve structures able to transmit mechanical deformation through electrical signals to dorsal root ganglion sensory neurons. Mechanoreceptor is a subtype of somatosensory receptor. It conveys extracellular stimuli through intracellular signal conduction via a mechanically gated ion channel. It conveys not only kinetic stimuli, but also pressure, stretching, touch, and even sound wave.

There are four types of MRCs classified by Freeman and Wyke [3]:

Type I: corpuscles of Ruffini—low-threshold, slowly adapting receptors that respond to mechanical stress. Ruffini endings appear to be stimulated by displacement of the collagen fibers with which they are intertwined. The characteristics of these receptors categorize them as static and dynamic mechanoreceptors, transmitting information about static position, changes in intra-articular pressure, and the direction, amplitude, and velocity of the joint movements.

Type II: corpuscles of Vater-Pacini—dynamic, rapidly adapting mechanoreceptors with a low threshold. They are entirely inactive in immobile joints, becoming active only at the onset or cessation of joint movement, moments at which sudden changes of stress occur.

Type III: corpuscles of Golgi—high threshold, slowly adapting mechanoreceptors that are completely inactive in immobile joints. They become active only in extreme ranges of movement and when considerable stress is generated in the joint.

Type IV: free nerve endings—high-threshold, non-adapting pain receptors.

Various histological methods have been used in identifying MRCs mostly using the gold chloride method [4,5]. Recently, immunological methods using specific antigen antibody reactions have been increasingly utilized [6,7]. Immunological methods are more reliable and easier to use compared to the traditional methods of histological staining. Histological staining methods most commonly identify the structurally normal MRCs only, while the immunological stains identify the functionally viable MRCs [7]. Three antibodies are widely used in immunohistochemical analysis of neuronal structures and have proven to be the most reliable method in the detection of MRCs: the polyclonal antibody against S-100, the one against p75 and the monoclonal antibody against PGP9.5 [8].

## 3. Anterior Cruciate Ligament (ACL)

The first histological demonstration of MRCs in human cruciate ligaments was performed by Schultz et al. (using gold-chloride staining techniques), taken at the time of amputation or autopsy [9]. They found 1–3 Golgi organs in each ligament, located at the surface of each ligament beneath the synovial membrane. Zimny et al. presented for the first-time a histological demonstration of two morphologically distinct MRCs in the human ACL, as they identified Ruffini and Pacinian corpuscles in 6 human subjects [4]. In 1998, Raunest et al. used a sheep model to investigate the presence of MRCs in cruciate ligaments using a gold-chloride method [10]. They found Pacinian corpuscles, Ruffini endings and Ruffini corpuscles. Under light microscopy, the Ruffini corpuscles were comparable to Golgi tendon organs, but smaller and generally appearing in cluster formations. Therefore, no Golgi tendon organ receptor was identified.

Using an immunocytochemical approach to identify nerve fibers and corpuscular endings, involving a monoclonal antibody directed against the 68-kDa neurofilament protein, Krauspe et al. found two types of corpuscular-like endings; “spiral-like” (type I) and “spray-like” (type II) endings in a child’s ACL mostly near its bony attachments [11]. Adachi et al. analyzed the changes of MRCs in the ruptured ACL remnants of 29 patients by gold chloride staining and reported that the MRCs in the ligament remnants could persist for a long time following an injury [12]. They found MRCs in all ACL remnants, most commonly subsynovially or on the superficial layer of the ligament. The median total number of MRCs in an ACL remnant was 18 (range 8–30), and the median density of mechanoreceptors was 25 (range 12–69)/g.

In recent studies, the most common performed technique alongside hemotoxin and eosin stains is immunohistological and immunofluorescence using specific antibodies to neurofilament elements, including S100 and neurofilament protein (NFP). Lee et al. harvested 36 tibial remnants during ACL reconstruction and 2 normal ACLs from healthy knees [13]. Nineteen MRCs (8 Ruffini, 11 Golgi) were identified in the two normal ACLs at both tibial and femoral attachments. In the remnant group, MRCs were observed in 12 out of 36 cases (33%), with a total of 17 MRCs (6 Ruffini and 11 Golgi) observed. The principal finding of this study was that the immunohistochemical staining method proved to be reliable and relevant in terms of specifically identifying the MRCs. Furthermore, the presence of Ruffini and Golgi at the tibial remnant of the ruptured ACLs and normal ACL substance was verified.

Dhillon et al. also evaluated the proprioceptive potential in residual ACL remnants using immunohistological methods [14]. A total of 63 ACL stumps were biopsied and evaluated using hemotoxin and eosin stains, and monoclonal antibodies to S-100 and NFP. Morphologically normal MRCs (hemotoxin and eosin) and proprioceptive fibers (positivity with monoclonal antibody for NFP) were found in 46% and 52.4% of stumps, respectively. Ruffini corpuscles, Pacinian corpuscles and Golgi-like organs were identified, mostly located subsynovially.

In their study, Sonnery-Cottet et al. investigated the histological features of the remaining fibers of 26 partial ACL tears. Immunohistochemical studies revealed numerous free nerve endings and few Ruffini and Golgi corpuscles [15]. Gao et al. investigated the morphology and quantity of MRCs in the remnant stumps of 40 injured ACLs using immunohistochemical methods [16]. Ruffini corpuscles, Pacinian corpuscles, Golgi-like tendon organs, and free nerve endings were observed in most of the ACL stump specimens examined in this study. Çabuk and Çabuk examined 4 tendons and 4 ligaments from 8 fresh frozen cadaveric knees using hematoxylin–eosin staining and immunohistochemistry (S100 immunostaining) [17]. In the quantitative analysis of the MRCs, free nerve endings were the predominant ones, followed by Ruffini and Golgi-like endings. No Pacini corpuscles were found. MRCs were located mostly near the bone insertions of the cruciate ligaments.

Sha et al. observed the survival condition and the quantitative variation of MRCs in the tibial remnant of the ruptured ACL in 60 human knees using immunohistochemistry staining with multiple primary antibodies [18]. The patients were divided into 4 groups according to the time from injury to surgery. Each group included 15 cases. The time duration between injury and surgery was less than 3 months, 3 to 6 months, 6 months to 1 year, and more than 1 year, in Group I, II, III, and IV, respectively. Six normal ACL specimens were taken from healthy knees amputated at thigh level due to trauma and were used as the control group. No significant difference was found among groups I–IV; however, the overall trend was for a decrease in the number of MRCs with the passage of time (33 in Group I, 23 in Group II, 24 in Group III, 13 in Group IV, and 13 in the normal control group, respectively). Additionally, 92 Ruffini-like corpuscles, 9 Pacini-like corpuscles, 5 unclassified neural endings and free nerve endings were also observed under light microscopy, but no Golgi organ-like corpuscles was identified. Nayak et al. harvested 38 injured native ACL stumps from patients undergoing ACL reconstruction and stained with neurofilament protein stain to detect functional MRCs [19]. Monoclonal antibody staining of nerve fibers was positive in 44.7% (17 out of 38) of the specimens. They found no association between duration of injury and presence of MRCs. Li et al. observed the changes in the quantity and morphology of MRCs in different-state remnant stumps of 57 ruptured ACLs [20]. A total of 2365 sections were subjected to immunofluorescence staining, and 147 Ruffini corpuscles, 40 Pacinian corpuscles, 8 Golgi-like tendon organs, and 58 atypical MRCs in all ligament specimens were identified. Free nerve endings were also observed, but were ignored due to their small size (Table 1).

## 4. Posterior Cruciate Ligament (PCL)

MRCs in PCL are almost equally evaluated in several studies. Katonis et al. were the first to investigate the existence of MRCs in the PCL of the healthy human knee joint [23]. Three types of nerve endings were observed. Ruffini’s corpuscles, Vater-Pacini corpuscles and free nerve endings, were mostly observed at each bony attachment. Franchi et al. obtained 9 PCLs from patients undergoing total knee arthroplasty and 5 PCLs from amputated limbs or fresh cadavers. Using the gold-chloride technique, they found all types of MRCs, and observed a remarkable decrease in the number of MRCs in patients with arthrosis [24].

Del Valle et al. obtained samples from 22 PCLs from patients undergoing total knee arthroplasty and 3 PCLs from cadaver specimens. Using immunohistochemical analysis with mouse monodonal antibodies against neurofilament protein (NFP), S-100 protein (S 100P), epithelial membrane antigen (EMA), and vimentin they found all the known MRCs apart from the Golgi-like ones [6]. Martins et al. analyzed samples of 34 PCLs from patients undergoing total knee arthroplasty using antibodies against the S-100 protein and neurofilaments. Immunomarking for neural structures was positive in 23 of the cases (67.5%) [25]. Specific mechanoreceptors were identified in 10 ligaments, with 9 type II cases (Pacini) and 6 type IV cases predominating. Again, no Golgi receptors were identified.

Cabuk et al. harvested PCLs from 30 patients undergoing total knee arthroplasty and 10 fresh frozen cadavers [26]. Using S100 immunostaining, they evaluated the number and type of MRCs. They found all the types of MRCs in both groups, but the most essential finding of their study was the reduction of them in patients with arthrosis. The only type in which no difference found was the Pacini corpuscles. Chun et al. obtained samples from 14 patients, who had undergone PCL reconstruction and 4 patients, who had undergone arthroscopic meniscal procedures only with an intact PCL [27]. Immunohistochemical studies were performed in order to identify the presence of MRCs. MRCs were present in 11 of 12 samples (91.7%) of the remnant PCL tissues and in all samples from the control group (Table 2).

## 5. Discussion

This literature review focuses on histological studies involving both cruciate ligaments. One of the most important findings is that both ligaments are extremely important in terms of proprioception, having a significant number of MRCs that communicate with the central nervous system. Recent immunohistochemical analyses demonstrated that almost all types of MRCs are present in cruciate ligaments. Furthermore, their total number is significantly higher compared to other ligaments and tendons of the knee joint, highlighting their crucial role in knee joint proprioception [17,28]. The absence of some types of MRCs in the lateral structures such as the lateral collateral ligament and the anterolateral ligament may also suggest the more important proprioceptive role of cruciate ligaments [29,30].

Free nerve endings are found most commonly, followed by Ruffini corpuscles, Pacinian corpuscles and Golgi-like tendon organs. Free nerve endings are mostly involved in the perception of pain and joint inflammation. Ruffini corpuscles are low-threshold, slowly adapting receptors that respond to mechanical stress. Any torque caused by extension, flexion and rotation of the joint can stimulate them to coordinate the overall movement of the knee and to feel position of the still knee joint. Pacinian corpuscles are low-threshold and rapidly adapted pressure receptors, play an important role in joint dynamic movement because they can produce and transmit joint motor sense and are very sensitive to the change of joint position. Golgi–like tendon organs are high-threshold and slow-adapted mechanoreceptors, which play an important role in preventing the joint from extreme flexion, extension and rotation. Of note, most of the MRCs are distributed in the synovium near the attachment points of ligaments with the femur and tibia, especially with the tibia. In a study of knee joint in rabbits, Han et al. found that cruciate ligaments had the higher number of MRCs in comparison with the other knee ligaments. Moreover, they found that gene expression level was positively related to the quantity of MRCs as the levels of NEFM, S-100B and CGRP genes were highest in ACL and PCL, suggesting that these ligaments have the richest sensory nerve endings [28].

Proprioception of the knee should always be taken into consideration for successful reconstruction of cruciate ligaments. Many studies have focused on the presence of MRCs in the torn ACL [5,12,13,14] and PCL stump, since the recovery of knee proprioceptive sense contributes to a successful CL reconstruction equally to the restoration of the knee mechanical stability. A major area of conflict is whether the presence of MRCs in the ligament remnants remains in time. There are studies showing that MRCs have no signs of degeneration [12,18], while others present that MRCs in the ligament remnants gradually decrease with the prolongation of injury time [5,14,16]. Most likely, the lack of specific analysis for ligament remnants explain that difference. A recent meta-analysis by Kosy et al. shows a decrease in MRCs in the remnants of the ruptured ACL with increasing time from rupture in multiple histological studies [31]. In a study of 2018 Li et al. found that there was no significant change in MRCs with a prolonged injury period in the ruptured ACL remnants that connected the femur to tibia and still played their roles in mechanical stability [20]. On the other hand, the MRCs number decreased gradually in ligament remnants with loss of mechanical functions.

Another important issue raised recently is the presence of MRCs in allografts and autografts used for cruciate ligament reconstruction. In 2020, Rebmann et al. studied 26 patients with different types of autografts (semitendinosus and patellar tendon) and three patients with patellar tendon allografts during revision surgery after traumatic rerupture of the ACL graft. Using immunohistochemical analysis, they found Ruffini corpuscles and free nerve endings present in each graft. Comparing the grafts, the highest number of MRCs could be detected in the semitendinosus autograft. With increasing time from implantation, the MRCs, especially in the patellar tendon autografts, showed an increase in their number for both Ruffini and free nerve endings. The authors concluded that the partial increase in the number of receptors over time after ACL reconstruction could indicate a reinnervation of the grafts [8]. Another study histologically evaluated the presence of mechanoreceptors in Achilles tendon allografts at a mean 26.63 months after ACL reconstruction. Remnant preservation techniques were not used. The specimens were obtained from the graft tendons superficially at the medial side of the femoral attachment, the midsubstance, and the tibial attachment, respectively, and stained with hematoxylin-eosin monoclonal antibodies against S-100. No mechanoreceptors were observed [32]. Chun et al. performed a similar study in 2017, but used improved immunocytochemical methodology and remnant preservation techniques. Neural cells were identified (although less frequently than expected) in both remnant ACL tissue and allografts. The authors state that “this finding does not negate the need for remnant-preserving ACL reconstruction. Indeed, the presence of mechanoreceptors is most important, and this should not be obscured by the limitations of current detection methodologies” [33].

Regarding knee arthrosis, proprioceptive acuity was proposed as a risk factor for the initiation and progression of pain and structural damage in knee [34]. In their study, Cabuk et al. found a reduction in the number of all types of MRCs in PCL of osteoarthritic knees except for the number of Pacini corpuscles [26]. This finding may explain the lack of correlation between the motion sensing and position sensing of the knee, as Pacini corpuscles function in motion sensing. Furthermore, in a very recent study, Al-Dadah et al. found that patients with isolated articular cartilage lesions of the knee had a significant proprioceptive deficit as compared to normal controls, which shows that articular cartilage lesions have a major influence on knee proprioception [35]. However, it remains uncertain as to whether a proprioceptive deficit leads to arthrosis or is a consequence of it.

As the number of total knee replacements (TKR) is expected to increase up to 143% by 2050 in the USA [36] and up to 91% by 2030 in Korea [37], it is fundamental for the orthopedic surgeon to reproduce the natural knee movement while maintaining stability in the whole range of movement. Frattura et al. found after a systematic review that proprioception in osteoarthritic patients undergoing TKR improves, but remains impaired after surgery [38]. One of the main issues in total knee arthroplasty is the retention or the sacrifice of PCL, as it seems to affect the proprioception of the knee. Bravi et al. concluded that the retention of the PCL does not substantially improve the joint proprioception after TKR [39].

## 6. Conclusions

Further research is required to fully understand the role of mechanoreceptors. Understanding the role of MRCs in knee kinematics should provide us with more information about the proprioceptive deficiencies associated with ligament ruptures and the pathogeneses of knee arthrosis. Patients at risk for MRCs-deficient knees could be candidates for special rehabilitation protocols, in order to compensate for loss of proprioception and kinesthesia.

## Figures and Tables

**Table 1:** Summary of ACL histological studies.

Study	Method	Findings
Shultz et al. 1984 [9]	Gold-chloride	1–3 Golgi organs
Zimny et al. 1986 [4]	Gold-chloride	Ruffini and Pacinian corpuscles
Schutte et al. 1987 [21]	Gold-chloride	Ruffini and Pacinian corpuscles, free nerve endings
Halata et al. 1989 [22]	Uranyl acetate and lead citrate	Ruffini and Pacinian corpuscles, free nerve endings
Krauspe et al. 1995 [11]	Ιmmunohistological	Ruffini and Pacinian corpuscles
Lee et al. 2009 [13]	Immunohistochemical staining	Ruffini and Golgi
Dhillon et al. 2010 [14]	Immunohistological and Hematoxin and Eosin	Ruffini corpuscles, Pacinian corpuscle and Golgi-like organs
Sonnery-Cottet et al. 2012 [15]	Immunohistochemical	Numerous free nerve endings and few Ruffini and Golgi corpuscles
Gao et al. 2016 [16]	Immunohistological and Hematoxin and Eosin	Ruffini corpuscles, Pacinian corpuscles, Golgi-like tendon organs
Çabuk and Çabuk 2016 [17]	Immunohistochemical	Ruffini, Golgi-like endings and free nerve endings
Sha et al. 2017 [18]	Immunohistochemistry staining	Ruffini-like corpuscles, Pacini-like corpuscles, unclassified neural endings and free nerve endings
Li et al. 2018 [20]	Immunofluorescence staining	Ruffini corpuscles, Pacinian corpuscles, Golgi-like tendon organs, atypical MRCs and free nerve endings

**Table 2:** Summary of PCL histological studies.

Study	Method	Findings
Shultz et al. 1984 [9]	Gold–chloride	1–3 Golgi organs
Katonis et al. 1991 [23]	Gold–chloride	Ruffini corpuscles, Vater-Pacini corpuscles and free nerve endings
Franchi et al. 1995 [24]	Gold–chloride	All types of MRCs
Del Valle et al. 1998 [6]	Immunohistochemical analysis	Ruffini corpuscles, Vater-Pacini corpuscles and free nerve endings
Martins et al. 2015 [25]	Immunohistochemical analysis	Ruffini corpuscles, Vater-Pacini corpuscles and free nerve endings
Cabuk and Cabuk 2016 [17]	Immunohistochemical analysis	Ruffini, Golgi-like endings and free nerve endings
Cabuk et al. 2017 [26]	Immunohistochemical analysis	All types of MRCS
Chun et al. 2020 [27]	Immunohistochemical analysis and Hematoxylin–Eosin	All types of MRCS

## Data Availability

Not applicable.
